# Solitary infantile myofibromatosis in the bones of the upper extremities: Two rare cases and a review of the literature

**DOI:** 10.3892/ol.2013.1584

**Published:** 2013-09-12

**Authors:** WEILIANG WU, JIANSONG CHEN, XINFANG CAO, MIN YANG, JIAN ZHU, GUOQIANG ZHAO

**Affiliations:** 1 Department of Orthopedics, Children’s Hospital, Zhejiang University School of Medicine, Hangzhou, Zhejiang 310003, P.R. China; 2 Department of Ophthalmology, The First Affiliated Hospital, Zhejiang University School of Medicine, Hangzhou, Zhejiang 310009, P.R. China; 3 Department of Pathology, Children’s Hospital, Zhejiang University School of Medicine, Hangzhou, Zhejiang 310003, P.R. China

**Keywords:** infantile myofibromatosis, solitary, ulna, humerus, extremities, bone

## Abstract

Infantile myofibromatosis (IM) is the most common fibrous tumor of infancy. IM may arise in a solitary or multicentric form, with similar histopathological findings, however, the clinical features and prognoses may vary. The solitary form tends to occur predominantly in males and is typically observed in the dermis, subcutis or deep soft tissues. The reported incidence of solitary osseous myofibromatosis is rare. Furthermore, the majority of solitary IM cases of the bone occur in the craniofacial bones, while the occurrence of solitary osseous myofibromatosis on the extremities has been sporadically reported. The present study describes two cases of solitary IM involving the bones of the upper extremities in females who were over two years old. The cases show unusual symptom presentation and the tumor origin is in a rarely observed location. The study discusses the clinical, radiological and pathological features, in addition to the previously described etiology, prognosis and treatment options for this condition.

## Introduction

Infantile myofibromatosis (IM), the most common fibrous tumor of infancy, is a mesenchymal disorder that is characterized by the proliferation of fibrous tumors in the skin, bone, muscle and viscera (1). The condition was first described by Stout in 1954 and was initially termed ‘congenital generalized fibromatosis’ (2). The tumor was renamed by Chung and Enzinger in 1981 to reflect its myofibroblastic characteristics (3). The World Health Organization (WHO), in the 2002 classification of soft tissue tumors, recognized myofibromatosis under the benign category of fibroblastic-myofibroblastic lesions (4). The condition usually presents prior to the age of two years (3), but may be observed in older children and even in adults (5). There are three distinct presentations, solitary, multicentric without visceral involvement and multicentric with visceral involvement. The solitary form tends to occur predominately in males (6) and is typically identified in the dermis, subcutis or deep soft tissues. The reported incidence of solitary osseous myofibromatosis is rare (7–9). The distribution is predominantly on the head, neck and torso, with only a rare involvement of the extremities (3). The present study describes two cases of solitary IM involving the bones of the upper extremities in females who were over two years old. The cases are unusual in their symptom presentation and the origin of the tumor is in a rarely observed location. Written informed consent was obtained from the patients.

## Case reports

### Case 1

**Figure 1:**
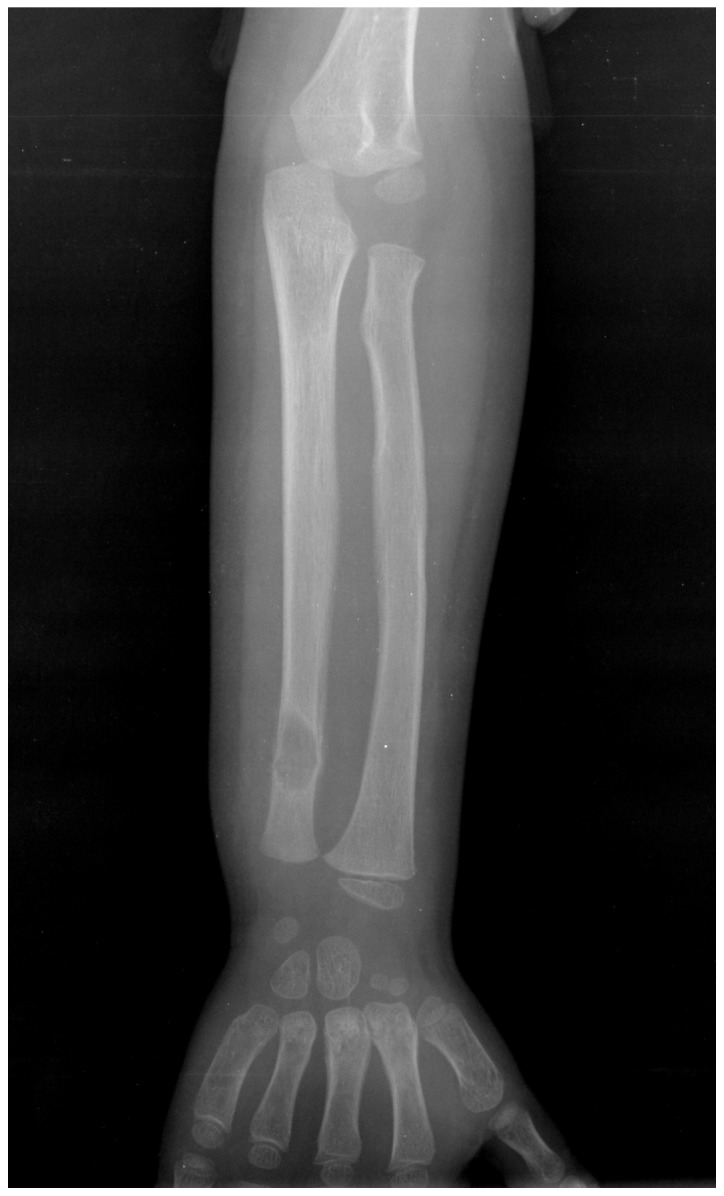
Case 1. X-ray demonstrating a well-circumscribed osteolytic lesion with slight marginal sclerosis involving the diaphysis of the distal left ulna.

**Figure 2:**
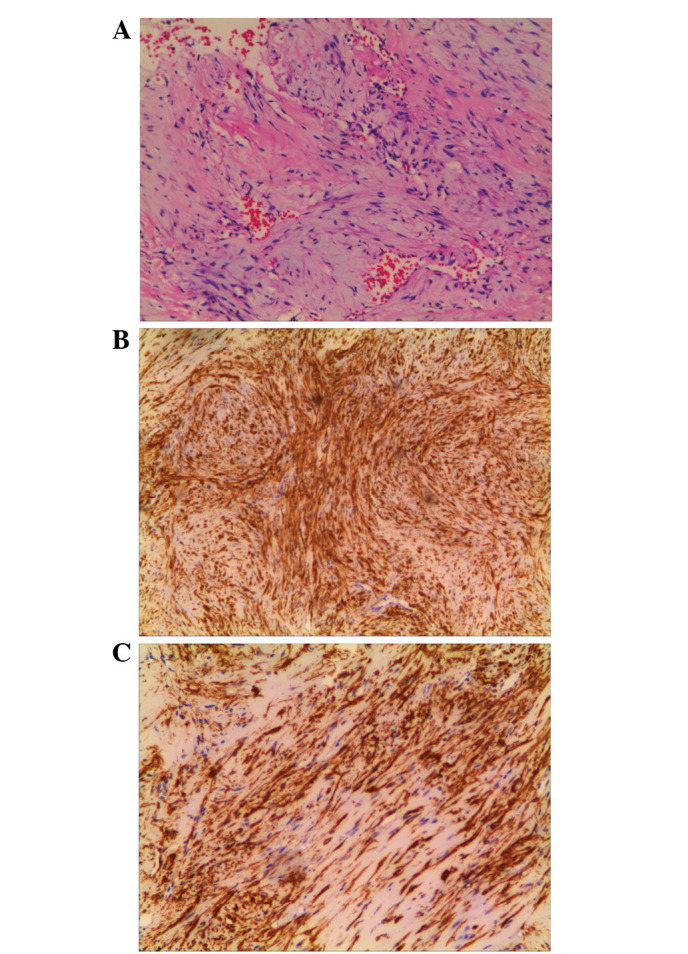
Case 1. (A) Photomicrograph exhibiting angiocentric and perivascular growth of myofibroblasts (HE staining; magnification, ×100). (B) Immunohistochemistry showing immunopositivity for smooth muscle actin (SMA; magnification, ×100). (C) Immunohistochemistry showing immunopositivity for vimentin (VIM; magnification, ×100).

A three-year-old female patient was admitted to Children’s Hospital of Zhejiang University School of Medicine (Hangzhou, China) with a 10-day history of enlargement of a lump located in the left forearm. The girl appeared systemically healthy with a firm, well-circumscribed, subcutaneous nodule situated in the left ulna and measuring 2×3 cm in dimension. The swelling was slightly tender, but the overlying skin was not inflamed and normal in color. The family history was unremarkable. An X-ray examination exhibited a well-defined osteolytic lesion with slight marginal sclerosis and a pathological fracture in the distal left ulna (Fig. 1). To rule out further tissue involvement, an ultrasound of the abdomen and a chest X-ray were performed, which provided negative results. The patient underwent curettage with bone grafting and flexible intramedullary nail fixation on February 1, 2012. Histologically, the specimen appeared to be formed of nodules that were composed of cytologically bland spindle cells and abundant hyalinized stroma. The cells were arranged in a fascicular and intertwining fashion with minimal mitotic activities and without pleomorphism or atypia. Blood vessels were abundant in a hemangiopericytoma-like pattern (Fig. 2A). Immunohistochemistry revealed positive staining for smooth muscle actin (SMA; Fig. 2B) and vimentin (VIM; Fig. 2C) and an absence of staining for desmin and S-100. A diagnosis of myofibromatosis was formed. There was no recurrence of any other lesions in the bone or soft tissues during a one-year follow-up period.

### Case 2

**Figure 3:**
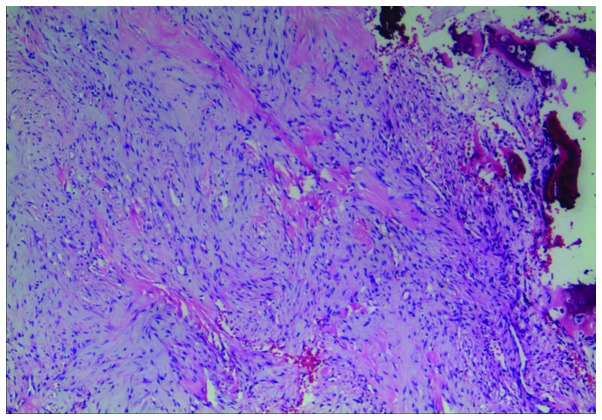
Case 2. Photomicrograph revealing biphasic cellular patterns and spindle cell proliferation (HE staining; magnification, ×40).

A nine-year-old female patient was sent to a local clinic following a fall. The radiological examination revealed a well-defined radiolucent lesion and a pathological fracture in the diaphysis of the right humerus. Subsequent to being transferred to Children’s Hospital of Zhejiang University School of Medicine for further treatment, the patient was in a good general condition, but was extremely vocal due to pain when the right arm was moved. The family and antenatal histories were unremarkable. The blood tests revealed a normal morphology and blood cell count. The laboratory values for calcium and phosphate were normal. Additional imaging studies failed to demonstrate any distant or visceral involvement. The patient underwent curettage with bone grafting and flexible intramedullary nail fixation on May 24, 2010. Macroscopically, fascicles of spindle cells with abundant eosinophilic cytoplasm resembled smooth muscle (Fig. 3). The spindle and plump cells stained positively for SMA and VIM, whereas desmin and S-100 markers were negative. The patient was histopathologically diagnosed with solitary myofibromatosis. The post-operative course was uneventful. During a two-year follow-up period, no recurrence was identified either locally or systemically.

## Discussion

IM is a fibrous tumor of childhood and infancy that is characterized by the development of nodular lesions involving the skin, subcutaneous tissue, internal organs or bones. The tumor may arise in a solitary or multicentric form, with similar histopathological findings, but varied clinical features and prognoses (5). Bone lesions are seldom observed with the solitary type (5%), but are common with the multicentric type (17–77%) (10–12). The majority of the solitary IM cases of the bone have occurred in the craniofacial bones (13). The occurrence of solitary osseous myofibromatosis of the extremities has been sporadically reported. When a review of the literature was performed, one case of solitary IM in the appendicular bone of the distal tibia was identified to have been reported by Inwards *et al*(14), one case in the distal ulna was reported by Kindblom and Angervall (15) and one case in the femur was reported by Yamamoto *et al*(13). In the two patients of the present study, the bone lesions of the solitary type involved the ulna and the humerus, respectively.

The etiology of this disease is not well understood. One of the proposed hypotheses is the upregulation of the estrogen receptors on the fetal smooth muscles, which leaves them more sensitive to maternal estrogen and may induce their proliferation (16). Two other patterns of inheritance have been described, autosomal dominant with low penetrance and autosomal recessive (17). Studies with regard to the specific genetic aberration have been limited, with monosomy 9q, trisomy 16q and del(6)(q12;q15) being the few cytogenetic abnormalities that have been reported (18,19).

The imaging characteristics of IM are not specific. On radiographic images, the bone lesions appear as osteolytic areas with sclerotic rims. On ultrasound scans, the masses may show either a hyperechoic or anechoic center with a surrounding rim. Computed tomography depicts the tumor as isodense or with a lower density compared with the muscles, and bone involvement often takes the shape of circumscribed lytic lesions with sclerotic margins (20). Magnetic resonance imaging of the tumor typically reveals a low intensity on T1-weighted images and a high intensity on T2-weighted images.

Histologically, the cells have a characteristic spindle shaped fibroblast appearance with pale pink cytoplasm and elongated nuclei when stained with HE. Calcifications are frequent observations and the mitotic activity is minimally increased (21). The typical IM immunohistological staining result is positive for VIM and SMA, whereas it is negative for S-100 epithelial membrane antigen and cytokeratin (22).

Myofibromas are frequently confused with a number of other entities, including benign and malignant leiomyoma, leiomyosarcoma, neurofibroma, fibrosarcoma, metastatic neuroblastoma, hemangiopericytoma, desmoplastic fibroma and inflammatory myofibroblastic tumors (23).

The prognosis of IM varies according to the type (24). Tumors without visceral involvement have an excellent outcome, with a spontaneous regression of the lesions in one to two years. By contrast, IM with visceral involvement is a severe disease. Gastrointestinal and cardiopulmonary complications determine the early morbidity and mortality of this IM presentation (24). Generally, the prognosis of solitary IM of the bone is favorable. Inwards *et al*(14) reported no recurrences during the post-operative follow-up of six cases. However, Kindblom and Angervall (15) reported a case of IM of the ulna that recurred twice following curettage. In the present study, no recurrence was identified either locally or systemically during the follow-up periods.

The treatment for IM is determined by the location of the lesion. Although spontaneous regression is reported in a large number of cases, recurrence has also been reported (10,11). Surgical excision should be reserved for cases that affect the vital functions (21). IM with visceral involvement may require surgical or medical treatment, including radiotherapy or chemotherapy with vincristine, actinomycin D and cyclophosphamide, together with supportive care (24). However, the literature is unclear on the overall success of these alternative methods (5). In the two patients of the present study, the bone lesions had resulted in pathological fractures and dysfunction of the forearms. The decision was ultimately reached to treat with complete local excision with bone grafting and flexible intramedullary nail fixation.

Although the incidence of solitary osseous myofibromatosis is rare, IM should be considered in the differential diagnosis for swellings and/or osteolytic lesions with pathological fractures in a child’s extremities. Subsequent to confirming a diagnosis, chest and abdominal imaging must be performed to evaluate the overall prognosis and to direct treatment (5). The patient should be followed up to assess recurrences and to exclude the manifestation of further nodules characterizing myofibromatosis (21).
